# Supplementary material for: Early Alterations of RNA Binding Protein (RBP) Homeostasis and ER Stress-Mediated Autophagy Contributes to Progressive Retinal Degeneration in the rd10 Mouse Model of Retinitis Pigmentosa (RP)
Source: Cells. 2023 Apr 6;12(7):1094. doi: 10.3390/cells12071094 (PMC10092976; doi:10.3390/cells12071094)
Supplement: Supplementary file 1 [file cells-12-01094-s001.zip › cells-2147176-supplementary.pdf]

cells

MDPI

Supplementary Material

Early Alterations of RNA Binding Protein (RBP) Homeostasis and ER Stress-Mediated Autophagy Contributes to Progressive Retinal Degeneration in the *rd10* Mouse Model of Retinitis Pigmentosa (RP)

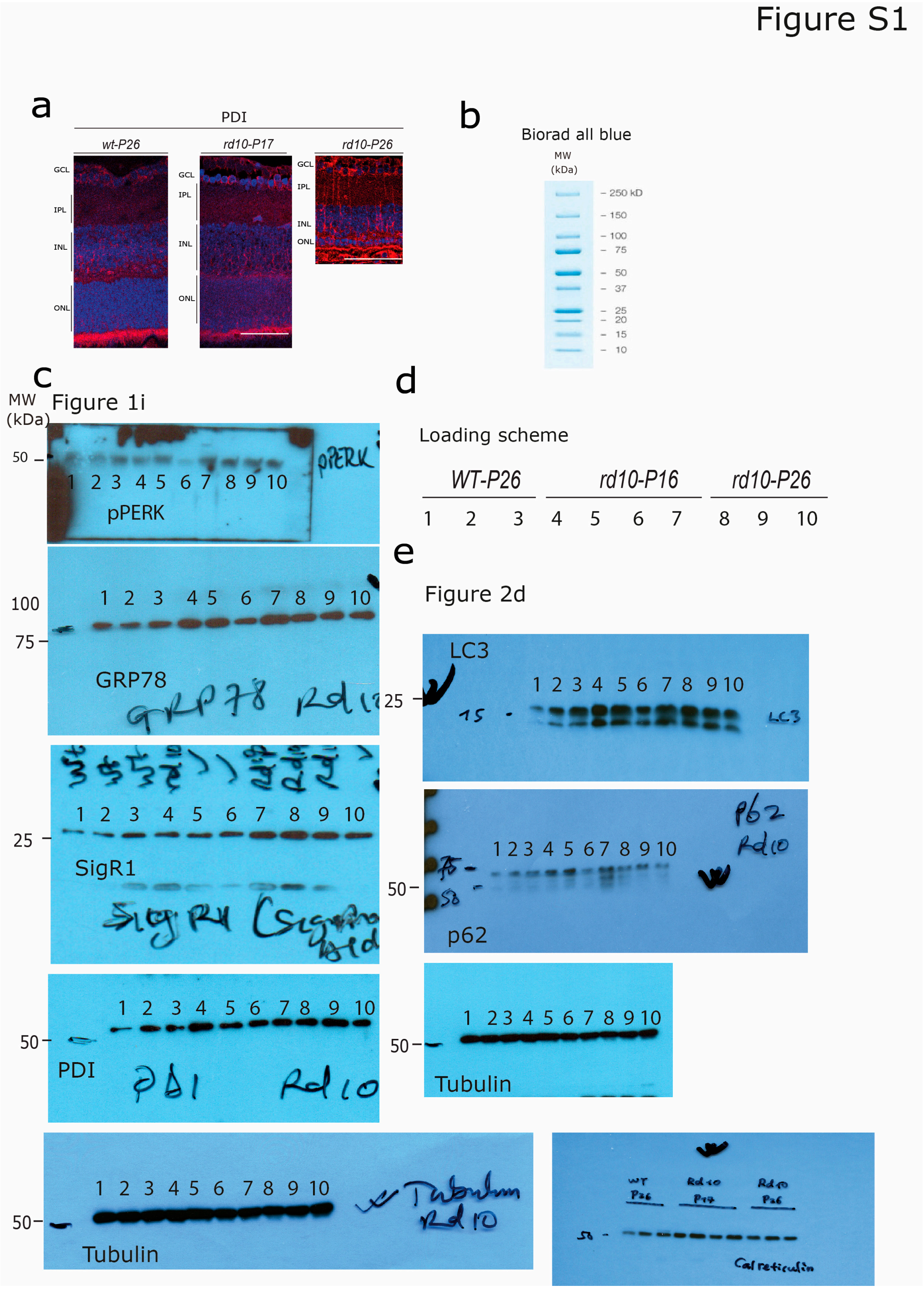

1

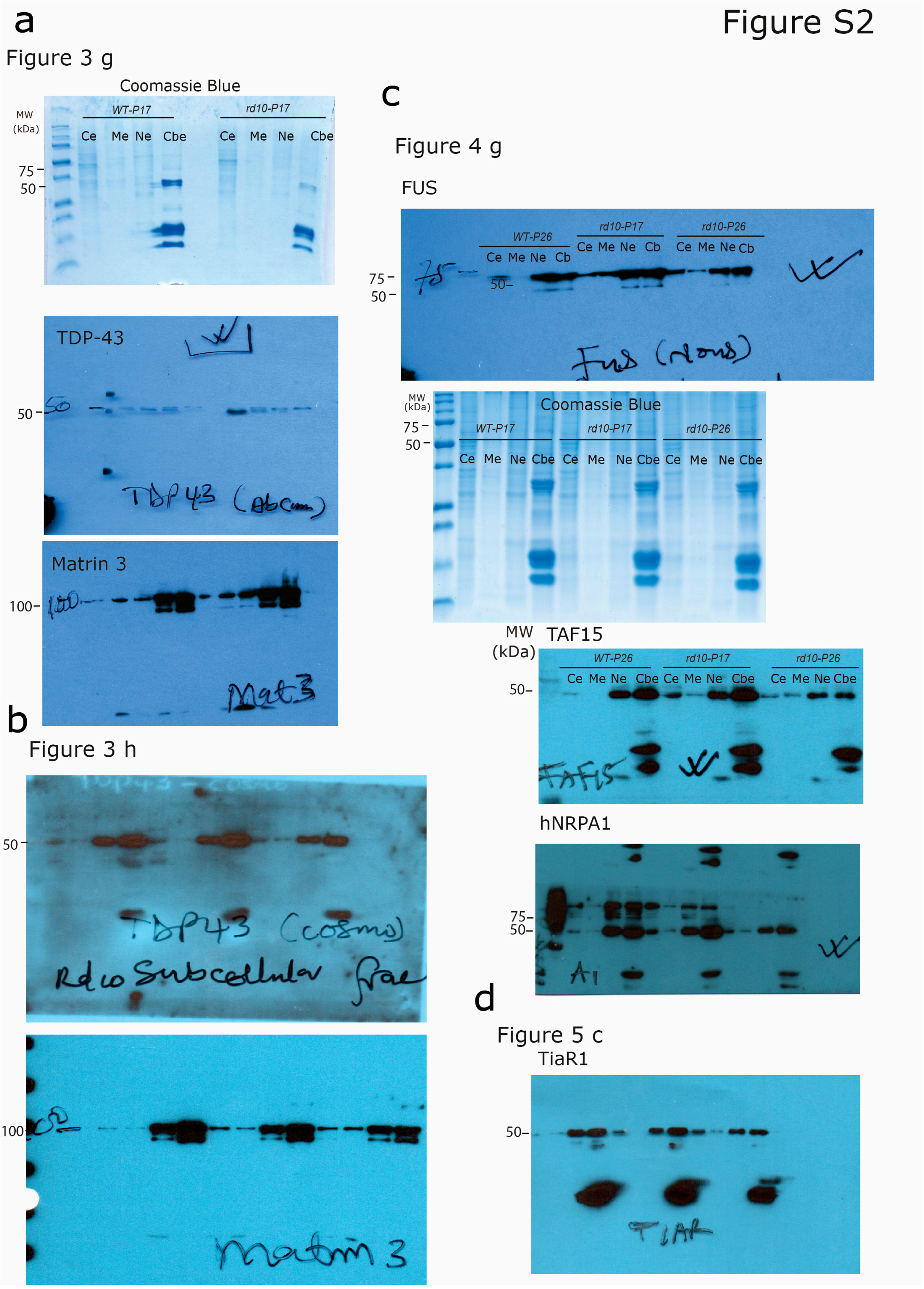
Figure S2
a
Figure 3 g
Coomassie Blue
MW (kDa)
WT-P17
rd10-P17
Ce Me Ne Cbe Ce Me Ne Cbe
75
50
TDP-43
50
Matrin 3
100
b
Figure 3 h
50
100
c
Figure 4 g
FUS
WT-P26
rd10-P17
rd10-P26
Ce Me Ne Cb Ce Me Ne Cb Ce Me Ne Cb
75
50
MW (kDa)
Coomassie Blue
WT-P17
rd10-P17
rd10-P26
Ce Me Ne Cbe Ce Me Ne Cbe Ce Me Ne Cbe
75
50
MW (kDa)
TAF15
WT-P26
rd10-P17
rd10-P26
Ce Me Ne Cbe Ce Me Ne Cbe Ce Me Ne Cbe
50
hNRPA1
75
50
d
Figure 5 c
TiaR1
50

Table S1. List of primary and secondary antibodies used in this study, previously used by us in the references below: [1–7].

Antibody	Commercial Source	Species	Reference	Working Dilution	
				IHC/IF	IB
**Primary antibody**					
Calreticulin (D3E6)	Cell signaling	Rabbit	12238S	1:150	
PKcα	Abcam	Mouse	ab86715	1:100	
Cabp	Sigma Aldrich	Mouse	C9848	1:100	
Cabp	Abcam	Rabbit	ab11246	1:100	
Anti-GRP78 (Bip)	BD Biosciences	Mouse	610978	1:100	1:1000
Anti-pPERK	Cell signalling	Rabbit	3179S	1:100	**1:1000**
Anti-PDI	Cell signalling	Rabbit	2446S	1:100	**1:1000**
Anti-GADD 153 (R-20)	Santa Cruz Biotech	Rabbit	SC-793	1:100	**-**
Anti-SigR1	Proteintech	Rabbit polycl	15168-1-AP	1:100	**-**
Anti-SigR1	Sigma Aldrich	Rabbit	HPA018002	1:100	1:1000
Anti-SigR1	Santa Cruz	Mouse	Sc-166392	1:50	1:1000
Anti-P62/SQSTM1	MBL	Rabbit	PM045	1:200	1:5000
Anti-P62/SQSTM1	Sigma Aldrich	Rabbit	P0067	1:100	1:5000
Anti-P62/SQSTM1	Abcam	Mouse	Ab56416	1:100	-
Anti-LC3	Sigma Aldrich	Rabbit	L7543	1:100	1:5000
Anti-pTDP43	Cosmo Bio Co. LTD	Mouse	TIP-PTD-M01	1:5000	**-**
Anti-TDP-43 C-terminus (405-414)	Cosmo Bio Co. LTD	Rabbit	TIP-TD-P09	-	1:100
Anti-TDP43	Abcam	Mouse	Ab57105	-	1:1000
Anti-Matrin3	Bethyl	Rabbit	IHC-00081	1:200	-
Anti-Matrin3	Bethyl	Rabbit	A300-591A	-	1:1000
Anti-Fus	Novus Biologicals	Rabbit	NB100-2599	1:100	1:1000
Anti-Fus	Sigma Aldrich	Mouse	AMAb90549	1:100	1:1000
Anti-TIAR	BD Biosciences	Mouse	610352	1:100	1:1000
Anti-EWSR1	Sigma Aldrich	Rabbit		1:100	1:1000
Anti-TAFII68	Bethyl	Rabbit	IHC-00094	1:100	1:1000
Anti-hnRNP A1	Novus Biologicals	Mouse	NB100-672	1:100	1:1000
Anti-hnRNP A2/B1	Santa Cruz Biotech	Mouse	SC-32316	1:100	1:1000
Anti α-Tubulin	Sigma Aldrich	Mouse	T5168	-	1:1000
**Secondary antibody**					
Poly HRP-GAMs/Rb IgG	Immunologica VWR	Ms/Rb	VWRKDPVB500HRP	Ready-to-use	
Poly HRP-Anti Goat IgG	Immunologica VWR	Goat	VWRKDPVG110HRP	Ready-to-use	

Alexa Fluor 488 goat anti-mouse	Life Technologies	Goat	A11001	1:500	
Alexa Fluor 555 goat anti-mouse	Life Technologies	Goat	A21424	1:500	
Alexa Fluor 488 goat anti-rabbit	Life Technologies	Goat	A11008	1:500	
Alexa Fluor 546 goat anti-rabbit	Life Technologies	Goat	A11010	1:500	
Alexa Fluor 488 donkey anti-goat	Life Technologies	Donkey	A11055	1:500	
Goat anti-mouse IgG (H+L), HRP	Thermo Scientific	Goat	31430		1:10000
Goat anti-rabbit IgG (H+L), HRP	Thermo Scientific	Goat	31460		1:10000

Table S2. Semi-quantitative analysis of DAB and immunofluorescence staining.

	**Wt-p26**			**Wt-p50**			**rd10-p17**			**rd10-p19**			**rd10-p26**			**rd-p50**		
	ONL	INL	GCL	ONL	INL	GCL	ONL	INL	GCL	ONL	INL	GCL	ONL	INL	GCL	ONL	INL	GCL
**Calcium binding/buffering chaperones**																		
calreticulin	+	+	+	+	+	+	++	+++	++	NA	NA	NA	+++	++	+++	NA	++	+
pPERK	+	+	+	+	+	+	++	+++	++	NA	NA	NA	+++	++	++	NA	NA	NA
PDI	+	+	+	+	+	+	++	+++	+++	NA	NA	NA	+++	+++	+++	NA	NA	NA
**ER stress markers**																		
grp78	+	+	+	+	+	+	++	++	++	NA	NA	NA	++	++	+++	NA	NA	NA
gadd-153	+	+	+	+	+	+	++	++	++	NA	NA	NA	++	++	++	NA	NA	NA
SigR1	+	+	+	+	+	+	++	++	++	NA	NA	NA	+++	++	++	NA	NA	NA
**Autophagy adapter/autophagosomes markers**																		
p62	+	+	+	+	+	+	++	+++	++●	NA	NA	NA	++	++●	++	NA	NA	NA
LC3	+	+	+	+	+	+	+++	+++	++	NA	NA	NA	++	+++	++	NA	++	++
**RNA binding proteins/stress granules**																		
pTDP43	+	+	+	+	+	+	+++●	+++●	++●	+++●	+++●	++●	++●	++●	++●	NA	++●	++●
Matrin 3	+	+	+	+	+	+	++	++	++	NA	NA	NA	++	++	++	NA	NA	NA
Fus	+	+	+	+	+	+	+++●	+++●	++●	+++●	++●	++●	++	++●	+●	NA	NA	NA
EWSR1	+	+	+	+	+	+	+++●	+++●	++●	++●	++●	++●	++●	++●	++●	NA	NA	NA
hnRNPA1	+	+	+	+	+	+	++●	+++●	++	NA	NA	NA	++●	++	++	NA	NA	NA
hnRNPA2B1	+	+	+	+	+	+	+++●	+++●	++●	NA	NA	NA	++●	++	++	NA	NA	NA
Tia1	+	+	+	+	+	+	+++●	+++●	++●	NA	NA	NA	++●	++	++	NA	NA	NA

+++ strong immunoreactivity/accumulation, ++ moderate immunoreactivity/accumulation, + mild immunoreactivity, ● aggregates, Not available, NA- not included in the study.

Table S3. Age group of wild-type/*rd10* mouse retinae and fixation protocols.

Experimental Methods	Age Group of Mouse Retinae (Left and Right) Wild-Type (C57BL/6)	Age Group of Mouse Retinae (Left and Right) *Rd10* (*C57BL/6J-Pde6b rd10/J*)	Fixation Protocol
Hematoxylin & Eosin staining (H&E)	P17, *n* = 3<br>P19, *n* = 3<br>P26, *n* = 3<br>P50, *n* = 3	P17, *n* = 3<br>P19, *n* = 3<br>P26, *n* = 3<br>P50, *n* = 3	4% PFA
Diaminobenzidine (DAB) Immunohistochemistry	P26, *n* = 3	P17, *n* = 3<br>P26, *n* = 3	4% PFA
Immunofluorescence	P17, *n* = 3<br>P26, *n* = 3<br>P50, *n* = 3	P17, *n* = 3<br>P26, *n* = 3<br>P50, *n* = 3	4% PFA
Transmission Electron Microscopy (TEM)		P19, *n* = 3<br>P22, *n* = 3	2.5% glutaraldehyde
Western Blot	P26, *n* = 3	P17, *n* = 4<br>P26, *n* = 3	Fresh<br>(No fixation)
Subcellular Fractionation	P17, *n* = 3<br>P26, *n* = 3	P17, *n* = 3<br>P26, *n* = 3	Fresh<br>(No fixation)
Filter Trap Assay	P26, *n* = 3	P17, *n* = 3<br>P26, *n* = 3	Fresh<br>(No fixation)

PFA: Paraformaldehyde.

References

1. Tripathi, P.; Guo, H.; Dreser, A.; Yamoah, A.; Sechi, A.; Jesse, C.M.; Katona, I.; Doukas, P.; Nikolin, S.; Ernst, S.; et al. Pathomechanisms of ALS8: Altered autophagy and defective RNA binding protein (RBP) homeostasis due to the VAPB P56S mutation. *Cell Death. Dis.* **2021**, *12*, 466. https://doi.org/10.1038/s41419-021-03710-y.
2. Yamoah, A.; Tripathi, P.; Sechi, A.; Kohler, C.; Guo, H.; Chandrasekar, A.; Nolte, K.W.; Wruck, C.J.; Katona, I.; Anink, J.; et al. Aggregates of RNA Binding Proteins and ER Chaperones Linked to Exosomes in Granulovacuolar Degeneration of the Alzheimer's Disease Brain. *J. Alzheimers Dis.* **2020**, *75*, 139–156. https://doi.org/10.3233/JAD-190722.
3. Dreser, A.; Vollrath, J.T.; Sechi, A.; Johann, S.; Roos, A.; Yamoah, A.; Katona, I.; Bohlega, S.; Wiemuth, D.; Tian, Y.; et al. The ALS-linked E102Q mutation in Sigma receptor-1 leads to ER stress-mediated defects in protein homeostasis and dysregulation of RNA-binding proteins. *Cell Death Differ.* **2017**, *24*, 1655–1671. https://doi.org/10.1038/cdd.2017.88.
4. Jesse, C.M.; Bushuven, E.; Tripathi, P.; Chandrasekar, A.; Simon, C.M.; Drepper, C.; Yamoah, A.; Dreser, A.; Katona, I.; Johann, S.; et al. ALS-Associated Endoplasmic Reticulum Proteins in Denervated Skeletal Muscle: Implications for Motor Neuron Disease Pathology. *Brain. Pathl.* **2017**, *27*, 781–794. https://doi.org/10.1111/bpa.12453..
5. Goswami, A.; Jesse, C.M.; Chandrasekar, A.; Bushuven, E.; Vollrath, J.T.; Dreser, A.; Katona, I.; Beyer, C.; Johann, S.; Feller, A.C.; et al.. Accumulation of STIM1 is asso-ciated with the degenerative muscle fibre phenotype in ALS and other neurogenic atrophies. *Neuropathol. Appl. Neurobiol.* **2015**, *41*, 304–318.

6. Vollrath, J.T.; Sechi, A.; Dreser, A.; Katona, I.; Wiemuth, D.; Vervoorts, J.; Dohmen, M.; Chandrasekar, A.; Prause, J.; Brauers, E.; et al.. Loss of function of the ALS protein SigR1 leads to ER pathology associated with defective autophagy and lipid raft disturbances. *Cell Death. Dis.* **2014**, *12*, 243.
7. Filezac de L'Etang, A.; Maharjan, N.; Cordeiro Brana, M.; Ruegsegger, C.; Rehmann, R.; Goswami, A.; Roos, A.; Troost, D.; Schneider, B.L.; Weis, J.; et al. Marinesco-Sjogren syndrome protein SIL1 regulates motor neuron subtype-selective ER stress in ALS. *Nat. Neurosci.* **2015**, *18*, 227–238.
